# Revision of the West Indian *Wattius* Kaszab (Tenebrionidae, Toxicini, Eudysantina) with lectotype designations for Pascoe’s South American species

**DOI:** 10.3897/zookeys.537.6115

**Published:** 2015-11-18

**Authors:** Aaron D. Smith, Lucio A. Sanchez

**Affiliations:** 1 Department of Biological Sciences, Northern Arizona University, PO Box 5640, Flagstaff, AZ, 86011-5640, USA

**Keywords:** Darkling beetles, new species, lectotype designation, Cuba, Bahamas, Dominican Republic

## Abstract

The *Wattius* species occurring in the West Indies are revised for the first time. *Wattius cucullatus* (Pascoe), previously reported from Cuba, is diagnosed and restricted to Brazil. *Wattius asperulus* (Pascoe), currently a synonym of *Wattius cucullatus*, from Colombia is diagnosed and **resurrected**. All species found in the West Indies are endemic to the islands and form a single informal species-group. Three species are described: *Wattius andersoni* **sp. n.** from Cuba, *Wattius emmabaconae* **sp. n.** from Hispaniola (Dominican Republic), and *Wattius viatorus* **sp. n.** from Cuba and the Bahamas, and lectotypes are designated for *Calymmus cucullatus* Pascoe and *Calymmus asperulus* Pascoe. A key to the West Indian species is provided.

## Abstract (es)

Las especies de *Wattius* que ocurren en las Indias Occidentales se revisan por primera vez. *Wattius cucullatus* (Pascoe), que se había registrado de Cuba, se diagnostica y se limita a Brasil. *Wattius asperulus* (Pascoe), actualmente sinónimo de *Wattius cucullatus*, se diagnostica y **es restaurado**, habitando en Colombia. Todas las especies que se encuentran en las Indias Occidentales son endémicas de las islas y forman un solo grupo informal de especies. Se describen tres especies: *Wattius andersoni* **sp. n.** de Cuba, *Wattius emmabaconae* **sp. n.** de Hispaniola (República Dominicana) y *Wattius viatorus* **sp. n.** de Cuba y las Bahamas. Se designan los lectotipos para *Calymmus cucullatus* Pascoe y *Calymmus asperulus* Pascoe. Se proporciona una clave de identificación para las especies que habitan las Indias Occidentales.

## Introduction

The New World component of the tribe Toxicini currently contains three genera, *Diceroderes* Solier (1 species, plus 4 species in press), *Ozolais* Pascoe (11 species), and *Wattius* Kaszab (5 extant species, one fossil), all in the subtribe Eudysantina Bouchard, Lawrence, Davies and Newton. *Wattius* was proposed by Kaszab (1982) as a name for the New World species previously in *Calymmus* Pascoe to separate them from the New Caledonian *Calymmus berardi* (Montrouzier, 1860). Hence, *Wattius* is restricted to the Americas and includes all known Eudysantina species with a single pronotal horn. Of the New World genera, all *Ozolais* species lack pronotal horns, but several species (including *Ozolais tuberculifera*) have one or two cephalic horns in the males; all *Diceroderes* have two pronotal horns; and all *Wattius* have a single pronotal horn that occurs in both sexes. The purpose of pronotal horns in the Eudysantina is still unclear. Label data indicates that most *Wattius* specimens have been collected by beating, under bark, or at UV lights. No specimens have been collected on fungus; however, Doyen (1988) described putative *Wattius cucullatus* (Pascoe, 1871) larvae collected from fungus, with no adults present, in Jalisco, Mexico.

**Figures 1–4.**
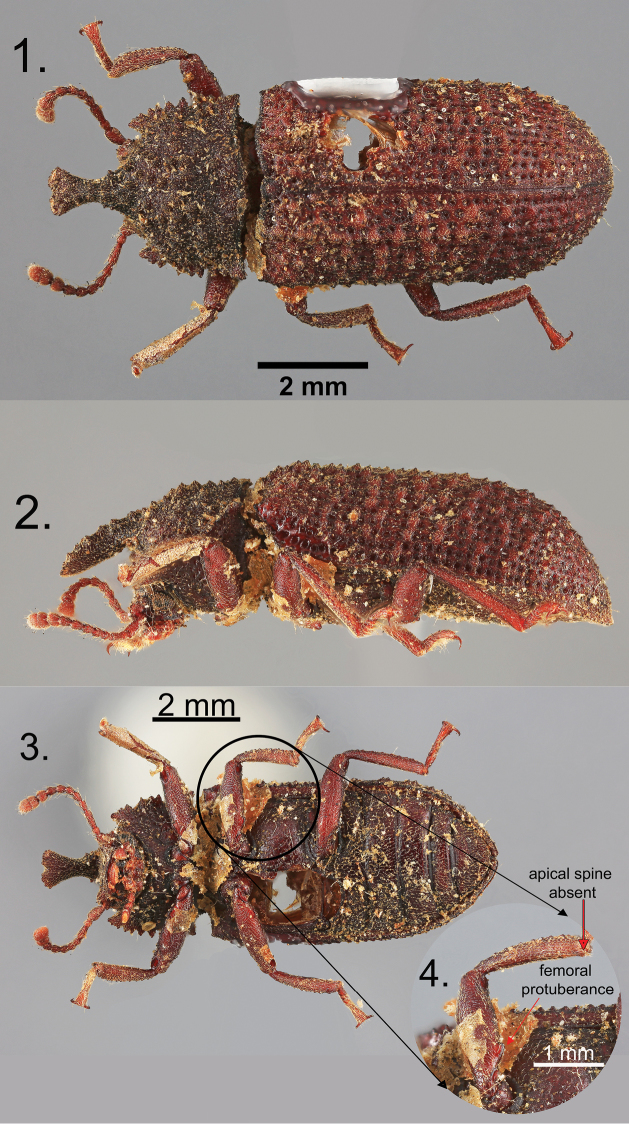
*Wattius cucullatus* (Pascoe), Lectotype. **1** Dorsal habitus **2** Lateral habitus **3** Ventral habitus **4** Close-up of meso-leg.

**Figures 5–6.**
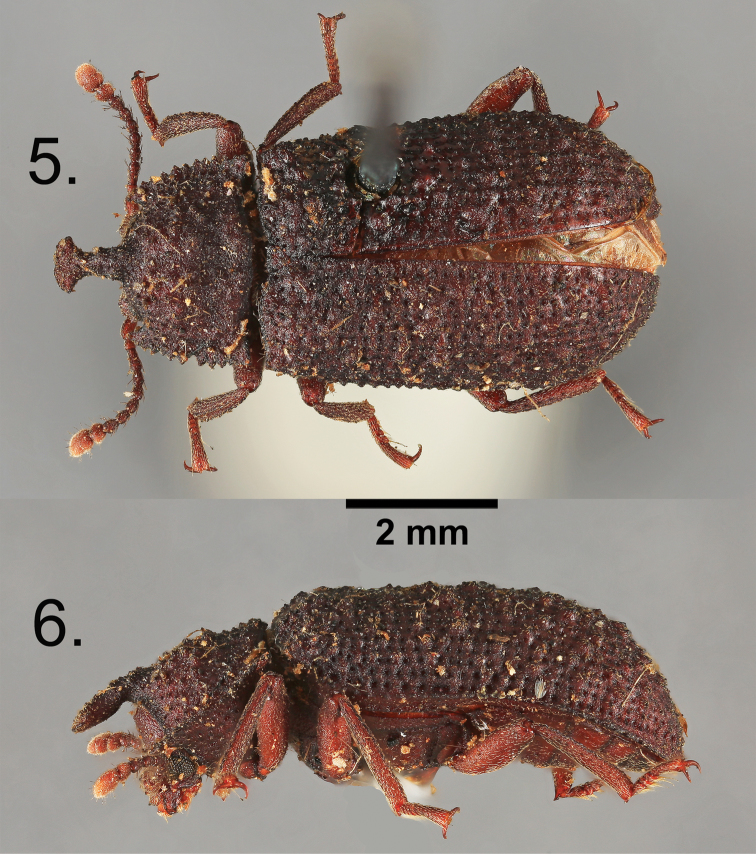
*Wattius asperulus* (Pascoe), Lectotype. **5** Dorsal habitus **6** Lateral habitus.

**Figure 7.**
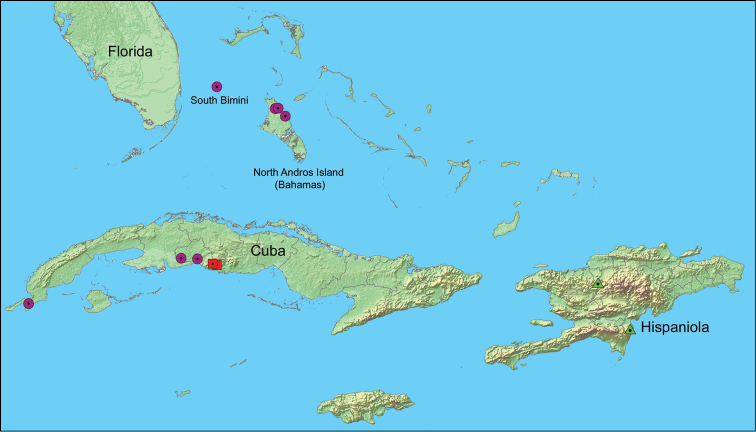
Distribution map of West Indian *Wattius* spp. Red squares = *Wattius andersoni* sp. n., green triangles = *Wattius emmabaconae* sp. n., purple circles = *Wattius viatorus* sp. n.

Two extant Toxicini species, *Ozolais tuberculifera* Champion, 1896 and *Wattius cucullatus* have so far been identified from the West Indies. *Wattius cucullatus* was recorded from Cuba (Spilman 1961, Peck 2005); however, all specimens were misidentified. Indeed, many specimens of *Wattius* from throughout the generic range have been mistakenly determined as *Wattius cucullatus* for over a century (Champion 1884, Cifuentes 2010), including the synonymization of *Wattius asperulus* (Pascoe, 1871) under *Wattius cucullatus* by Champion (1886). However, based on an examination of Pascoe’s syntype (Figures 1–4) from the Natural History Museum – London and over 600 *Wattius* specimens from 13 collections, specimens attributable to the true *Wattius cucullatus* have only been identified from the Brazilian states of Santa Catarina and Rio de Janeiro (type locality). This species is so far unique in the genus for having rounded protuberances on the posterior surface of all femora in the males (Figure 4). *Wattius asperulus* (Pascoe, 1871), previously and incorrectly placed in synonymy with *Wattius cucullatus*, from Colombia is here resurrected based on an examination of the syntype (Figures 5–6). After studying all available material, three undescribed endemic species are now known to occur in the West Indies (Figure 7) and are described herein.

One fossil species, *Wattius reflexus* Doyen & Poinar, 1994, has been described from Dominican amber, and additional amber specimens potentially representing an undescribed species have been found (Smith and Poinar, unpublished data). There is still uncertainty regarding the age of Dominican amber (Grimaldi 1995, Grimaldi and Engel 2005), especially when the source mine is unknown, but *Wattius* was almost certainly present on Hispaniola somewhere between 40–15 million years ago (mya).

## Materials and methods

A total of 56 West Indian *Wattius* specimens from 13 collections were examined for this study, along with ~635 non-West Indian specimens in the genus. Labels for new type material described herein are given verbatim in quotes, with lines separated by a forward slash. Individual labels are indicated alphabetically from uppermost to bottommost label. Specimens used in this study were graciously loaned to the first author from the following collections.

AMNH American Museum of Natural History, New York, NY, USA. (Lee Herman)

BMNH The Natural History Museum, London, United Kingdom. (Max Barclay)

CASC California Academy of Sciences, San Francisco, CA, USA. (Norm Penny)

CUIC Cornell University Insect Collection, Ithaca, NY, USA. (Jason Dombroskie) CMNC – Canadian Museum of Nature, Ottawa, Canada. (Bob Anderson)

EMEC Essig Museum of Entomology, University of California, Berkeley, CA, USA. (Peter Oboyski)

FMNH Field Museum of Natural History, Chicago, IL, USA. (Margaret Thayer)

HNHM Hungarian Natural History Museum, Budapest, Hungary. (Otto Merkl)

MNHN Muséum National d’Histoire Naturelle, Paris, France (Antoine Mantilleri)

WIBF West Indian Beetle Fauna Project, Montana State University, Bozeman, MT, USA. (Michael Ivie)

OSUC C. A. Triplehorn Insect Collection, Ohio State University, Columbus, Ohio, USA. (Charles A. Triplehorn)

SEMC Snow Entomological Museum, University of Kansas, Lawrence, KS, USA.

USNM National Museum of Natural History, Smithsonian Institution, Washington, DC, USA. (Warren Steiner)

ZMHB Museum für Naturkunde der Humboldt-Universität, Berlin, Germany (Bernd Jaeger)

ZSMC Zoologische Staatssammlung München, Munich, Germany (Martin Baehr)

Label data from all specimens was captured and additional specimen information, including images and determined GPS coordinates, is available online (tenebrioniDBase.org) for many specimens.

**Morphological parameters.** Images of specimens and/or morphological characters and character states were taken using a BK Plus Imaging system (www.visionarydigital.com). Montaged images were assembled using Helicon Focus 5.3 (www.heliconsoft.com/) and backgrounds were cleaned in Adobe Photoshop CS6. Measurements were taken digitally using the ruler tool in Photoshop on images with known measurements based on the camera body, lens, and magnification used. Length was measured along the midline from the anterior margin of the pronotum, generally the tip of the pronotal horn, to the apex of the elytra. Width was measured across the widest point of the elytra. Color was determined under fiber optic illumination and from images. Setae are acuminate and defined as either simple (circular in cross section) or scale-like (thickened, flattened in cross section). Density of recurring features (primarily punctures, tubercles, and setae) and puncture size closely are as follows. Density: confluent (partially merged), dense (separated by between less than 1–2 feature diameters but not partially merging), moderate (separated by 2–4 feature diameters), sparse (separated by more than 4 feature diameters), or absent (impunctate, smooth, or glabrous). Puncture size: foveate (rounded pits, diameter greater than 0.04 mm), moderate (diameter 0.03–0.04 mm), or fine (diameter less than 0.03 mm).

*Wattius* specimens are almost always encrusted in a waxy shellac-like exudate. The encrusting can be flaked off with a low gage pin, then wiped away with a camel hair brush. Cleaning specimens in soapy water and an ultrasonic cleaner also works but in some instances a waxy coating reformed upon drying.

**Species recognition.** The phylogenetic species concept of Wheeler and Platnick (2000) is employed to define a species as “the smallest aggregation of (sexual) populations or (asexual) lineages diagnosable by a unique combination of character states”. This species concept is appropriate due to its emphasis on character transformations between species and the lack of available data beyond adult morphology and distribution. Species were erected based on the presence of autapomorphic morphological characters and/or a unique combination of homoplastic characters shared by all of the specimens assigned to a species. Recognized species should be considered as scientific hypotheses based on the available data and, as such, their validity can be tested as more information is gathered.

## Results

The island species form a morphologically distinct group, the informal West Indian species-group, within the genus based on the lighter coloration of the antennal club (generally yellowish) compared to the other antennomeres, the glabrous and impunctate scutellum that is often relatively free of debris compared to the rest of the body, and the lack of depressions or other modifications on the frons.

### Key to the West Indian species of *Wattius* Kaszab

**Table:** 

1	Apterous, metaventrite length less than metacoxal cavity length; pronotal horn reduced (Figs 8–9); mentum with weakly raised, indistinct medial longitudinal ridge; Cuba	***Wattius andersoni* sp. n.**
–	Macropterous, metaventrite length greater than metacoxal cavity length; pronotal horn well developed (Figs 10–17); mentum with distinct medial longitudinal ridge, projecting anteriorly; Cuba, Bahamas, Hispaniola	**2**
2(1)	Scutellum triangular (Fig. 10); frons with shallow fovea and sharp tubercles between apex of eye and clypeus; femora with raised smooth callosities; males lacking apical tibial spine (Figs 4, 10–11); Hispaniola	***Wattius emmabaconae* sp. n.**
–	Scutellum U-shaped to weakly pentagonal (Fig. 16); frons with deep fovea, lacking sharp tubercles between apex of eye and clypeus; femora without raised callosities; males with apical tibial spine on all legs; Cuba and Bahamas	***Wattius viatorus* sp. n.**

**Figures 8–9.**
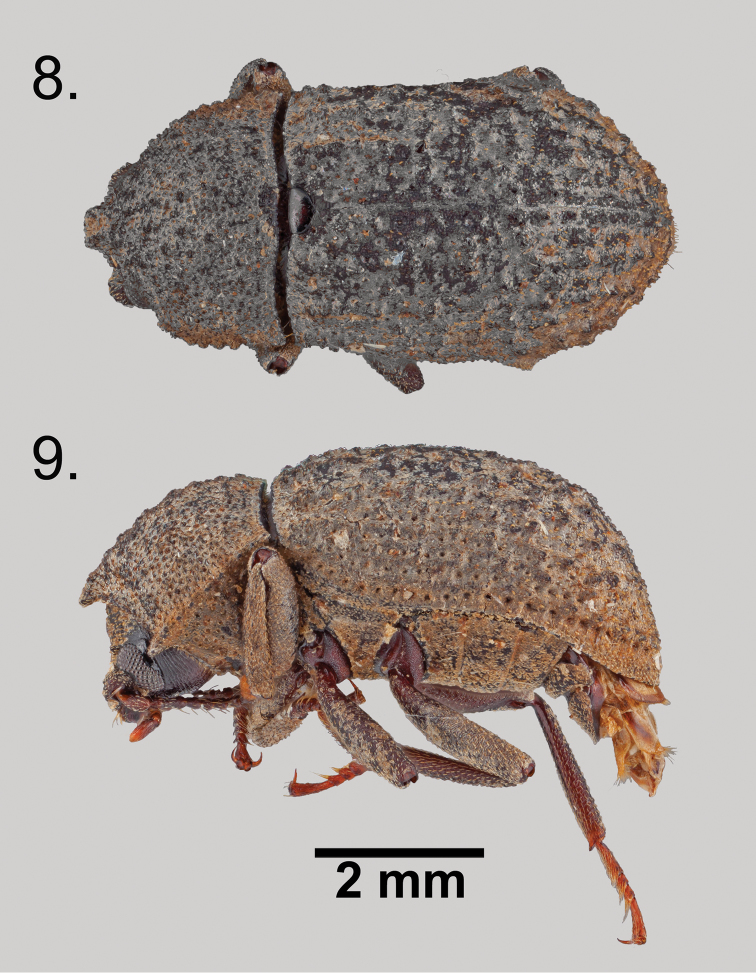
*Wattius andersoni* sp. n., Holotype (male). **8** Dorsal habitus **9** Lateral habitus.

**Figures 10–11.**
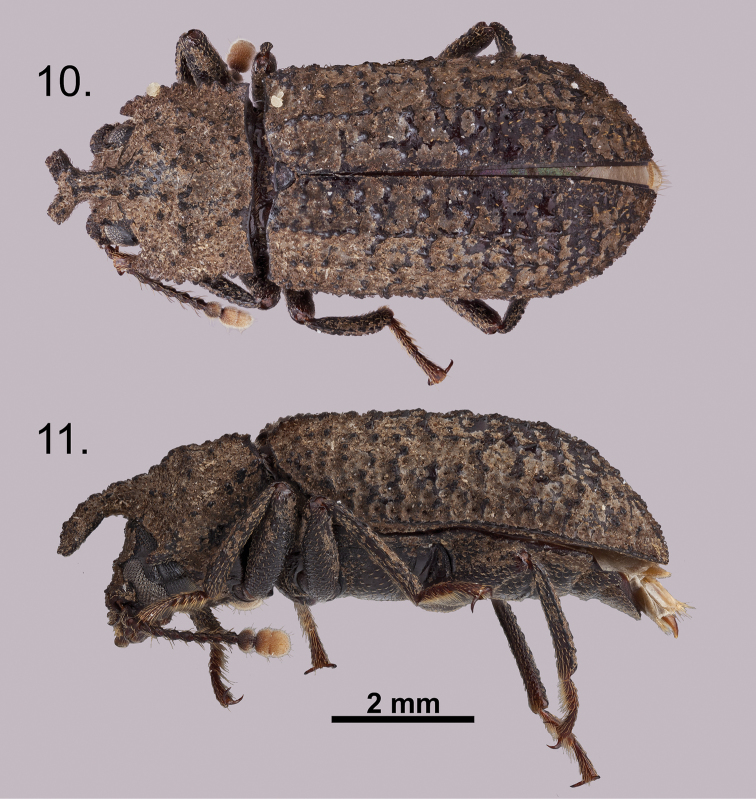
*Wattius emmabaconae* sp. n., Holotype (male). **10** Dorsal habitus **11** Lateral habitus.

**Figures 12–13.**
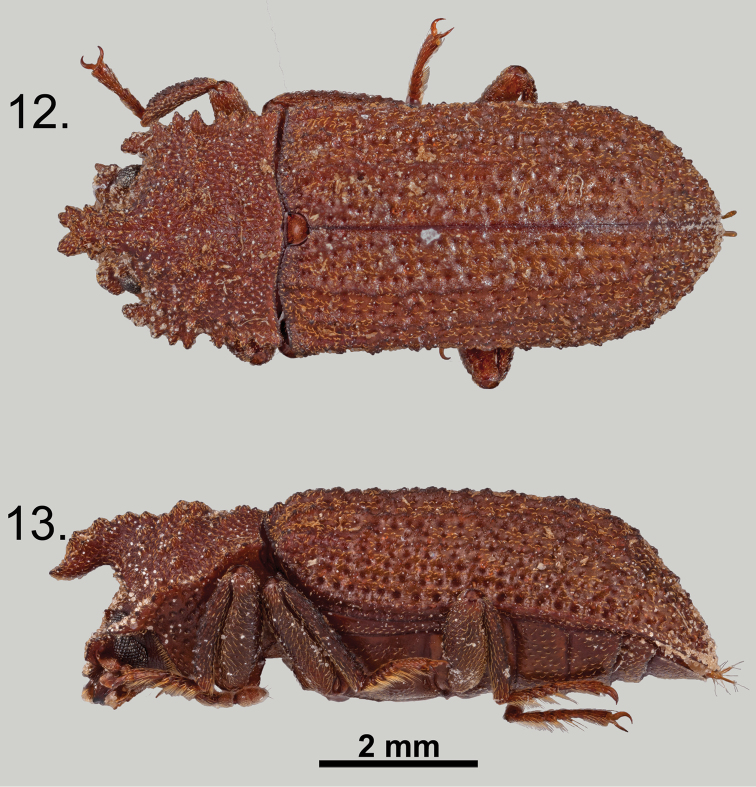
*Wattius emmabaconae* sp. n., Allotype (female). **12** Dorsal habitus **13** Lateral habitus.

**Figures 14–15.**
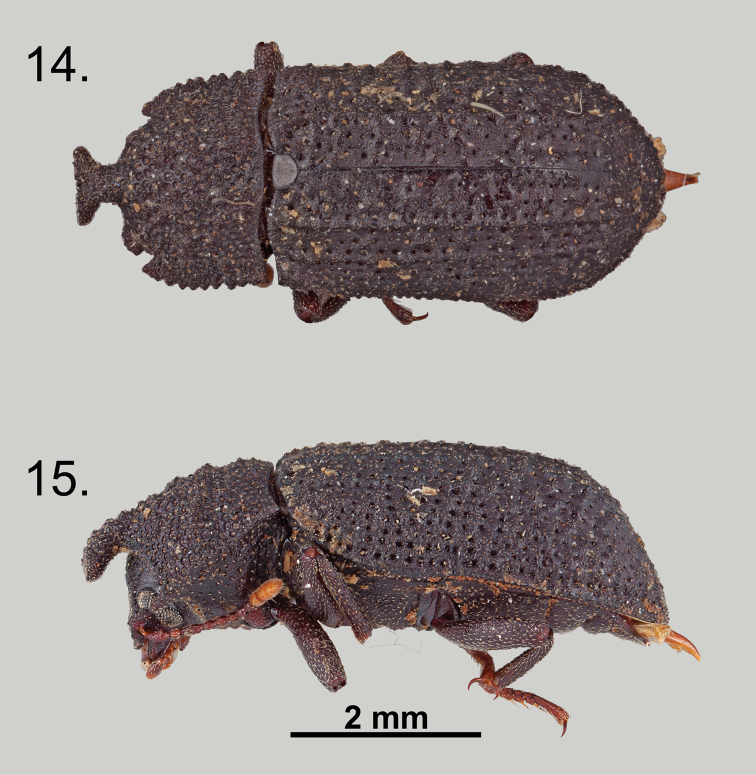
*Wattius viatorus* sp. n., Holotype (male). **14** Dorsal habitus **15** Lateral habitus.

**Figures 16–17.**
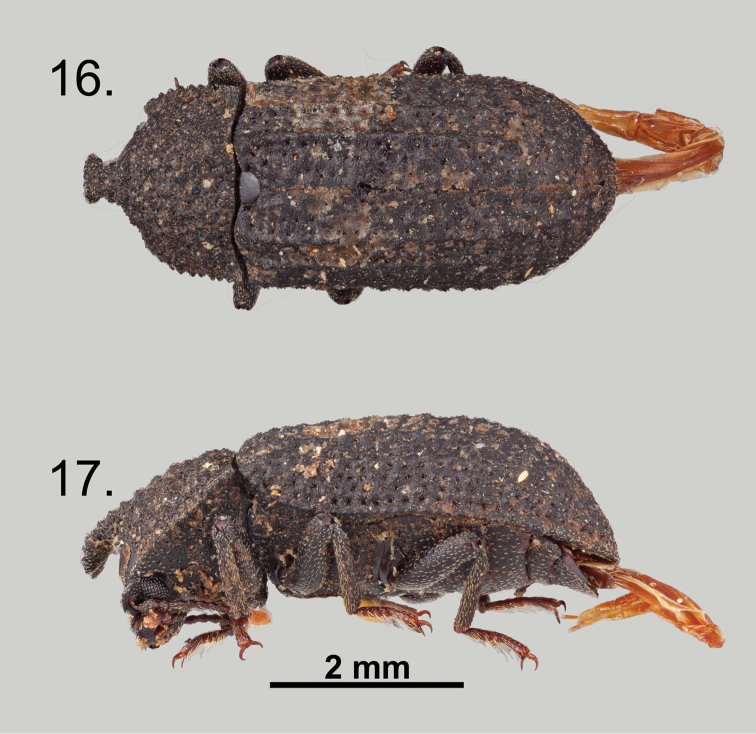
*Wattius viatorus* sp. n., Allotype (female). **16** Dorsal habitus **17** Lateral habitus.

### Clave para las especies de *Wattius* Kaszab del Indias Occidentales

**Table:** 

1	Áptero, longitud del metaventrito menor que la longitud de la cavidad metacoxal; cuerno pronotal reducido (Figs 8–9); mentón con cresta longitudinal medial ligeramente elevada; Cub	***Wattius andersoni* sp. n.**
–	Macróptero, longitud del metaventrito mayor que la longitud de la cavidad metacoxal; cuerno pronotal bien desarrollado (Figs 10–17); mentón con cresta longitudinal medial muy elevada, proyectada hacia anterior; Cuba, Bahamas, Hispaniola	**2**
2(1)	Escutelo triangular (Fig. 10); frente con fóvea superficial y tubérculos agudos entre el ápice del ojo y el clípeo; fémures con callosidades lisas y elevadas; better: tibias del macho sin espina apical (Figs 4, 10–11); Hispaniola	***Wattius emmabaconae* sp. n.**
–	Escutelo en forma de U a débilmente pentagonal (Fig. 16); frente con fóvea profunda, sin tubérculos agudos entre el ápice del ojo y el clípeo; fémures sin callosidades elevadas; todas las tibias del macho con una espina apical; Cuba and Bahamas	***Wattius viatorus* sp. n.**

## Species descriptions and diagnoses

### Wattius cucullatus

Taxon classificationAnimaliaColeopteraTenebrionidae

(Pascoe)

Figures 1–4

Calymmus cucullatus Pascoe, 1871: 349
Calymmus asperulus Pascoe, 1871: 350; synonymized in Champion 1886: 225
Wattius cucullatus (Pascoe); Kaszab 1982: 50

#### Type material.

LECTOTYPE, here designated, (male) labeled: on pink oval (a) “Rio”; (b) “Calymmus / cucullatus / Type Pasc”; (c) on white circle with orange border “Type”; (d) “Pascoe. / Coll. / 93–60.”; (e) on blue paper “Tenebrionid Base / Aaron D. Smith / Catalog # 14794”; (f) on red paper, “LECTOTYPE / Calymmus / cucullatus / Pascoe / det. ADSmith 2015” (BMNH).

#### Note.

Specimen designated as lectotype appears to be the one figured in the original publication (Pascoe 1871). No indication of the number of specimens in the original type series is given; however, it is possible that this was not the only individual.

#### Additional material examined.

Thirteen specimens. Six labeled “BRAZIL: Sta. Catarina / Nova Teutonia / [dates from Jan. 1975, Nov. 1977, and Mar. 1977] / F. Plaumann” (CASC - 3♂, 2♀, EMEC - 1♂). Five labeled “Lanca / St. Cath. Brazil / Oct. 1944” (AMNH - 1♂, 4♀ ). One female labeled “Cauna / S.Cath., Brazil / Dec. 1948” (AMNH). One female labeled “Rio Vermelho / S.Cath., Brazil / I. 1946” (AMNH).

#### Diagnosis.

*Wattius cucullatus* can be separated from all other known members of the genus based on the following character combination: Frontoclypeal suture strongly incised, frons shallowly depressed anterior to eyes; pronotal horn strongly produced, apex weakly bifurcated in males, prosternal process deflexed behind coxae, rarely with small projecting tubercle near apex; flight wings fully developed, meso- and metacoxae separated by more than mesocoxal width; femora lacking smooth rounded callosities; outer margins of tibia flat with two indistinct rows of callosities, apical spine absent on all tibia in males, all male femora with rounded protuberance on anterior edge of basal half.

### Wattius asperulus

Taxon classificationAnimaliaColeopteraTenebrionidae

(Pascoe) new status

Figures 5–6

Calymmus asperulus Pascoe, 1871: 350

#### Type material.

LECTOTYPE, here designated, (male) labeled: on pink oval (a) “Colombia”; (b) “Calymmus / asperulus / Type Pasc”; (c) on white circle with orange border “Type”; (d) “Pascoe. / Coll. / 93–60.”; (e) on blue paper “Tenebrionid Base / Aaron D. Smith / Catalog # 14322”; (f) on red paper, “LECTOTYPE / Calymmus / asperulus Pascoe / det. ADSmith 2015” (BMNH).

#### Additional material examined.

Two specimens, both from Colombia. One female labeled “P’to. Berrio / Ant. Colomb” (FMNH) and one male labeled “Colombia” (HNHM).

#### Diagnosis.

*Wattius asperulus* can be separated from *Wattius cucullatus* and the West Indian members of the genus based on the following character combination: Frontoclypeal suture strongly incised, frons shallowly depressed anterior to eyes and raised into near vertical ridge above eyes to cranial apex; pronotal horn strongly produced, apex expanded and spatulate in males, prosternal process raised behind coxae; wings fully developed, meso- and metacoxae separated by more than mesocoxal width; femora lacking smooth rounded callosities; outer margins of tibia flat with two indistinct rows of callosities, apical spine present on all tibia in males, all male femora lacking rounded protuberances.

#### Discussion.

*Wattius asperulus* was synonymized under *Wattius cucullatus* by Champion (1886). Champion’s rationale was that the separation between *Wattius cucullatus* and *Wattius asperulus* could not be maintained based on the variability displayed in the specimens, which ranged from Mexico to Argentina, available to him. However, a large series of BMNH specimens identified as *Wattius cucullatus* by Champion, and corresponding to many of the localities listed in the Biologia Centrali-Americana, represents an estimated seven *Wattius* species that are only now being described (Smith in prep.).

### Wattius andersoni

Taxon classificationAnimaliaColeopteraTenebrionidae

Smith & Sanchez sp. n.

http://zoobank.org/BEACFF8C-FC13-4C3B-8707-BFE54973D0F5

Figures 7 , 8–9

#### Type material.

HOLOTYPE (male) labeled: (a) “CUBA: Cienfuegos / Mayari, 2 km E. / 21.96651 -80.11497, 842m / 18.v.2013, R.Anderson / 2013-017X, hardwood forest”; (b) “WORLD / WEEVIL / DATABASE / WWD0104144”; (c) “Tenebrionid Base / Aaron D. Smith / Catalog # 14681”; (d) on red paper, “HOLOTYPE / Wattius / andersoni / Smith & Sanchez 2015” (CMNC). PARATYPE (male) labeled: (a) “CUBA: Topes de Col- / lantes, Sierra de / Trinidad, I.V. prov. / June 11, 1959”; (b) “M. Wattius Sanderson / C59-25”; (c) “ Tenebrionid Base / Aaron D. Smith / Catalog # 14154”; (d) on yellow paper, “PARATYPE / Wattius / andersoni / Smith & Sanchez 2015” (CMNC).

#### Diagnosis.

*Wattius andersoni* can be separated from the other West Indian members of the genus based on the following character combination: apterous, meso- and metacoxae separated by less than mesocoxal width; pronotal horn reduced, barely projecting past medial anterior margin of pronotum.

#### Description

**(Male).** Length 5.4–5.7 mm, width 2.5–2.7 mm (n = 2 specimens). Body, excepting antennae, eyes, underside of head, scutellum, tarsi, and coxae generally coated with thin shellac, often capturing debris on surface. Color ferruginous to black. **Head**: Frons and clypeus with dense foveae, shallow to absent on clypeus, each fovea with one decumbent scale-like setae near center. Sharp setose tubercle with minute pit at apex present above eye, setae curved towards tubercle apex. Frontoclypeal suture distinct, deeply impressed; clypeus with sharp lip along anterior margin, margin straight. Epistoma between eye and clypeus raised, with one or more tubercles. Deep

impression present around eye from epistoma to apex. Eye reniform; emarginate at epistoma anteriorly, lobes subequal in size, with smooth triangular callus posterior to dorsal lobe on head. Labrum with transverse medial ridge, long golden setae present from ridge to anterior margin on dorsal surface, margin straight with lateral setae on vertical surface. Mandible bifid at apex; maxillary palp four segmented, apical segment securiform; mentum trapezoidal, widest at anterior margin, faint medial longitudinal ridge present, more defined in anterior half. Antenna with distinct three segmented club, club lighter than preceding segments and tomentose, antennomeres 10 and 11 partially fused but with sinus clearly visible; antennomere 3 approximately 1.7× length of antennomere 4, antennomeres 4–8 subequal in length. **Prothorax**: Pronotal disc convex, widest near middle; densely foveate, each fovea with one decumbent scale-like setae near center; moderately tuberculate, each tubercle bearing apical minute pit and covered in scale-like setae curved towards apex; anterior fourth of pronotum with short stout medial horn, horn margin straight; posterior fourth of pronotum with slight medial depression near scutellum; lateral margin distinct and crenulate; anterior apices produced and acute, posterior apices acute, not projecting. Hypomeron densely deeply foveate, each fovea with one decumbent scale-like setae. Prosternum anterior to coxa short, less than length of coxal cavity, medially depressed well below height of prosternal process; prosternal process raised between coxa, apex subacute, projecting behind coxa. **Pterothorax**: Apterous. Elytron gradually widening to posterior third, before sharply sloping and tapering caudad; stria weakly indicated by deep elongate oval to rounded punctures, interstria with somewhat regularly spaced tubercles and decumbent scale-like setae, tubercle structure as described for those on head and pronotum; 4th, 7th, and 10th interstria with tubercles forming short costae near elytral base. Scutellum glabrous and impunctate, conspicuously lacking shellac coating compared to elytron and pronotum, ~1.6× as wide as long, U-shaped. Mesoventrite short, anteriorly weakly emarginate behind prosternal process, mesocoxal cavities open. Metaventrite short, separating meso- and metacoxal cavities by less than mesocoxal cavity length. All ventrites on the pterothorax with shallow indistinct punctures, often obscured by shellac, and decembent scale-like setae. **Legs**: Mesotrocantin exposed; femora lacking spines or other protrusions, sculpturing finely transversely rugose, with decumbent scale-like setae emerging from shallow folds; tibia clothed in decumbent scale-like setae, outer margins with indistinct rows of elongate smooth callosities, inner apical margin with socketed spurs greatly reduced to absent at base of acute weakly curved spine, small patch of golden setae present near anterior apex of protibia; tarsal formula 5-5-4, venter of distal tarsomere on all legs with sparse golden setae, venter of all other tarsomeres clothed with dense long golden setae. **Abdomen**: Ventrites weakly longitudinally rugose, clothed in sparse decumbent scale-like setae; abdominal intercoxal process broader than prosternal process, anterior margin straight; intersegmental membranes concealed; ventrite 5 lacking submarginal groove; abdominal defensive reservoirs present; sternite viii weakly sclerotized and setose, deeply medially emarginate, emargination V-shaped; parameres fused, sharply acuminate to apex and curved ventrad.

**Female.** Unknown. Based on an examination of other species in the genus, the female is likely to be very similar to the male, except lacking apical spines on the tibia and emargination on sternite viii.

#### Distribution.

Both specimens were found above 750m in elevation in the Parque Natural Topes de Collantes, Cuba.

#### Etymology.

The species epithet honors Robert S. Anderson, weevil expert, avid field researcher, and collector of the holotype.

### Wattius emmabaconae

Taxon classificationAnimaliaColeopteraTenebrionidae

Smith & Sanchez sp. n.

http://zoobank.org/DCD9DB2E-0D6B-4A69-BB10-691C6D34D822

Figures 7 , 10–11 , 12–13

#### Type material.

HOLOTYPE (male) labeled: (a) “DOMINICAN REP: Prov / Barahona, nr.Filipinas. / Mt. Tutu; 26-VI-7-VII- / 1992; P.E. Skelley / day catch, beating”; (b) “OSUC 524311”; (c) “Tenebrionid Base / Aaron D. Smith / Catalog # 13781”; (d) on red paper, “HOLOTYPE/ Wattius / emmabaconae / Smith & Sanchez 2015”. (OSUC). ALLOTYPE (female) labeled: (a) “DOMINICAN REPUBLIC/ Dajabon Prov. Los Cerezos./ 14km NW of Rio Limpio / 608-Fresh cut wood/ 19°18’42.9”N 71°36’36.6” W/ 29 June 2010 S.Lingerfelter”; (b) “Tenebrionid Base / Aaron D. Smith / Catalog # 13780”; (c) on red paper, “ALLOTYPE / Wattius / emmabaconae / Smith & Sanchez”. (SEMC) Paratype (male) labeled: (a) “DOMINICAN Rep. :Prov / Barahona. nr. Filipinas. / Larimar Mine: 26-VI-7- / VII-1992: Woodruff & / Skelly. at light”; (b) “OSUC 524312”; (c) “Tenebrionid Base / Aaron D. Smith / Catalog # 13779”; (d) on yellow paper, ‘PARATYPE / Wattius / emmabaconae / Smith & Sanchez 2015”. (OSUC)

#### Diagnosis.

*Wattius emmabaconae* can be separated from the other West Indian members of the genus based on the following character combination: flight wings fully developed, meso- and metacoxae separated by more than mesocoxal width; pronotal horn strongly produced, apex strongly expanded and bifurcate in males; femora with smooth rounded callosities; outer margins of tibia with two distinct rows of elongate smooth callosities, males lacking apical spine.

#### Description

**(Male).** Length 8.0–8.4 mm, width 2.8–3.3 mm (n = 2 specimens). Body, excepting antennae, eyes, underside of head, scutellum, tarsi, and coxae generally coated with thin shellac, often capturing debris on surface. Color ferruginous to black. **Head**: Frons and clypeus with dense shallow foveae, shallow to absent on clypeus, each fovea with one decumbent scale-like setae near center. Sharp setose tubercle with minute pit at apex present above eye, setae curved towards tubercle apex; one or more similar tubercles present between apex of eye and frontoclypeal margin. Frontoclypeal suture distinct, deeply impressed; clypeus with sharp lip along anterior margin, margin straight. Epistoma between eye and clypeus raised, with one or more sharp tubercles. Deep impression present around eye from epistoma to apex. Eye reniform; emarginate at epistoma anteriorly, ventral lobe larger than dorsal, with micro-granulate triangular callus posterior to middle of eye. Labrum with transverse medial ridge, long golden setae present from ridge to anterior margin on dorsal surface, margin straight with setae on vertical surface. Mandible bifid at apex; maxillary palp four segmented, apical segment securiform; mentum trapezoidal, widest at anterior margin, medial longitudinal ridge present, forming anteriorly facing tooth near front margin. Antenna with distinct three segmented club, club lighter than preceding segments and tomentose, antennomeres 10 and 11 fused, with sinus visible near lateral edges, segment 9 darker at base and lightening towards apex and lateral margins; antennomere 3 approximately 1.2× length of antennomere 4, antennomeres 4–8 subequal in length. **Prothorax**: Pronotal disc weakly convex, widest anterior to middle; densely, nearly confluently, shallowly foveate, each fovea with one decumbent scale-like setae near center; moderately tuberculate, forming irregular V-shaped pattern from near scutellum to anterior fourth, each tubercle bearing apical minute pit and covered in scale-like setae curved towards apex; anterior fourth of pronotum giving rise to raised medial horn, horn gradually sloping towards head, strongly expanded and bifid in apical third of length; posterior fourth of pronotum with slight medial depression, lacking tubercles, near scutellum; lateral margin distinct and crenulate; anterior apices strongly produced and acute, posterior apices acute, not projecting. Hypomeron densely shallowly foveate, each fovea with one decumbent scale-like setae. Prosternum anterior to coxa approximately as long as coxal cavity, medially nearly level with prosternal process; prosternal process raised between coxa, apex acute, projecting behind coxa. **Pterothorax**: Wings fully developed. Elytron parallel sided to posterior fourth, before sharply sloping and tapering caudad; stria weakly indicated by deep rounded punctures, interstria with somewhat regularly spaced tubercles and decumbent scale-like setae, tubercle structure as described for those on head and pronotum; 4th, 7th, and 10th interstria with tubercles forming weak costae, tubercles between 4th and 7th interstria and elytron suture occasionally forming irregular transverse costae. Scutellum glabrous and impunctate, width approximately 1.4× length, nearly V-shaped. Mesoventrite short, sparsely setose, anteriorly weakly emarginate behind prosternal process with submedial rows of rounded tubercles anterior to mesocoxal cavities, mesocoxal cavities open. Metaventrite long, separating meso- and metacoxal cavities by more than mesocoxal cavity length, sparsely setose with decumbent scale-like setae, impunctate. All other ventrites on the pterothorax micro-granulate, often obscured by shellac, with decembent scale-like setae. **Legs**: Mesotrocantin exposed; femora lacking spines or other protrusions, sculpturing finely transversely rugose with irregular smooth callosities on distal laterad half, decumbent scale-like setae emerging from shallow folds throughout; tibia clothed in decumbent scale-like setae, outer margins with two distinct rows of elongate smooth callosities, inner apical margin with socketed spurs and apical spine vestigial to absent, small patch of golden setae present near anterior apex of all tibia; tarsal formula 5-5-4, venter of distal tarsomere on all legs with sparse golden setae, venter of all other tarsomeres clothed with dense long golden setae. **Abdomen**: Ventrites smooth, clothed in sparse decumbent scale-like setae; abdominal intercoxal process subequal in width to prosternal process, anterior margin with small medial projection; intersegmental membranes concealed; ventrite 5 lacking submarginal groove; abdominal defensive reservoirs present; sternite viii weakly sclerotized and setose, deeply medially emarginate, emargination V-shaped; parameres fused, weakly acuminate to apex and curved ventrad.

**Female.** Similar to male, horn very weakly expanded and bifid at apex.

#### Distribution.

Known from only two localities in the Dominican Republic on Hispaniola.

#### Etymology.

The specific epithet is in honor of Emma C. Bacon and was chosen by her loving partner Christiaan Harden, a generous contributor to the authors’ ongoing biodiversity studies.

### Wattius viatorus

Taxon classificationAnimaliaColeopteraTenebrionidae

Smith & Sanchez sp. n.

http://zoobank.org/7E9D769E-76D0-4BDF-A1EE-78B7C2D2ED71

Figures 7 , 14–15 , 16–17

#### Type material.

HOLOTYPE (male) labeled: (a) “Cayamas / 29.5 Cuba”; (b) “EASchwarz / Collector”; (c) “Tenebrionid Base / Aaron D. Smith / Catalog # 14184”; (d) on red paper “HOLOTYPE / Wattius / viatorus / Smith & Sanchez 2015” (USNM). ALLOTYPE (female) labeled: (a) “Cayamas / 23.5 Cuba”; (b) “EASchwarz / Collector”; (c) “Tenebrionid Base / Aaron D. Smith / Catalog # 14183”; (d) On red paper “ALLOTYPE / Wattius / viatorus / Smith & Sanchez 2015” (USNM). PARATYPES (49 specimens) (all bearing the label “PARATYPE / Wattius / viatorus / Smith & Sanchez 2015” on yellow paper and the database label “Tenebrionid Base / Aaron D. Smith / Catalog # ”, for convenience tenebrioniDBase catalog numbers are listed as TB# without quotations). PARATYPE (female) labeled: (a) “Cayamas / 24.5 Cuba”; (b) “EASchwarz / Collector”; (c) TB# 14155; (USNM). PARATYPE (male) labeled: (a) “Cayamas / 25.5 Cuba”; (b) “EASchwarz / Collector”; (c) TB# 14156; (USNM). PARATYPE (male) labeled: (a) “Cayamas / 12.3 Cuba”; (b) “EASchwarz / Collector”; (c) TB # 14157; (USNM). PARATYPE (male) labeled: (a) “Cayamas / 7.2 Cuba”; (b) “EASchwarz / Collector”; (c) TB # 14158; (USNM). PARATYPE (male) labeled: (a) “Cayamas / 26.2 Cuba”; (b) “EASchwarz / Collector”; (c) TB # 14159; (USNM). PARATYPE (male) labeled: (a) “Cayamas / 8.5 Cuba”; (b) “EASchwarz / Collector”; (c) TB# 14160; (USNM). PARATYPE (male) labeled: (a) “Cayamas / 8.5 Cuba”; (b) “EASchwarz / Collector”; (c) TB# 14161; (USNM). PARATYPE (female) labeled: (a) “Cayamas / 10.2 Cuba”; (b) “EASchwarz / Collector”; (c) TB# 14162; (USNM). PARATYPE (male) labeled: (a) “Cayamas / 10.6 Cuba”; (b) “EASchwarz / Collector”; (c) TB# 14163; (USNM). PARATYPE (male) labeled: (a) “Cayamas / 11.3 Cuba”; (b) “EASchwarz / Collector”; (c) TB # 14164”; (USNM). PARATYPE (male) labeled: (a) “Cayamas / 15.3 Cuba”; (b) “EASchwarz / Collector”; (c) TB# 14165; (USNM). PARATYPE (male) labeled: (a) “Cayamas / 15.3 Cuba”; (b) “EASchwarz / Collector”; (c) TB # 14166; (USNM). PARATYPE (female) labeled: (a) “Cayamas / Cuba, Baker”; (b) “4175”; (c) TB# 14167; (USNM). PARATYPE (male) labeled: (a) “BAHAMAS: ANDROS ID. / London Ridge, 2.7 mi. N., / 0.8 mi. E., Forfar Field /Stn.,30.IV.1994-012, / R.S. Anderson, high / interior coppice”; (b) TB # 14169; (WIBF). PARATYPE (female) labeled: (a) “BAHAMAS: ANDROS ID. / London Ridge, 2.7 mi. N., / 0.8 mi. E., Forfar Field /Stn.,30.IV.1994-012, / R.S. Anderson, high / interior coppice”; (b) TB # 14170; (WIBF). PARATYPE (female) labeled: (a) “BAHAMAS: ANDROS ID. / London Ridge, 2.7 mi. N., / 0.8 mi. E., Forfar Field /Stn.,30.IV.1994-012, / R.S. Anderson, high / interior coppice”; (b) TB # 14171; (WIBF). PARATYPE (female) labeled: (a) “BAHAMA IS; N. Andros Is. / Atala Coppice, 10 km WNW / Stafford Creek town / 13.VI.1983”; (b) “Collector: / B.D. Valentine / and family”; (c) “OSUC 524309”; (d) TB # 14172. PARATYPE (male) labeled: (a) ”Cienfuegos / 3-22-39 CUBA / J.C. Biddley”; (b) TB # 14173; (CUIC). PARATYPE (female) labeled (a) “BAHAMAS: ANDROS ID. / Fresh Creek, Androsia / Factory, 26.IV.1994-006 / R.S. Anderson beating / interior dry coppice”; (b) TB # 14168; (WIBF). PARATYPE (male) labeled (a) “South Bimini Isl. / Bahamas,B.W.I. / August 15, 1951 / C.& P.Vaurie”; (b) TB # 14175; (AMNH). PARATYPE (male) labeled (a) “BAHAMAS: ANDROS ID. / London Ridge, 2.7 mi. N., / 0.8 mi. E., Forfar Field / Stn.,30.IV.1994-012, / R.S. Anderson, high / interior coppice”; (b) TB # 14179; (WIBF). PARATYPE (male) labeled (a) “BAHAMAS: ANDROS ID. / London Ridge, 2.7 mi. N., / 0.8 mi. E., Forfar Field / Stn.,30.IV.1994-012, / R.S. Anderson, high / interior coppice”; (b) TB # 14179; (WIBF). PARATYPE (female) labeled (a) “BAHAMAS: ANDROS ID. / London Ridge, 2.7 mi. N., / 0.8 mi. E., Forfar Field Stn., / 5-6.V.1994, R.S. Anderson, / high interior coppice beating”; (b) TB # 14180; (WIBF). PARATYPE (female) labeled (a) “South Bimini Isl. / Bahamas, B.W.I. / July 23,1951 / C. & P. Vaurie”; (b) TB # 13782.; (AMNH). PARATYPE (female) labeled (a) “South Bimini Isl. / Bahamas, B.W.I. / August 15,1951 / C. & P. Vaurie”; (b) TB# 14176.; (AMNH). PARATYPE (male) labeled (a) “South Bimini Isl. / Bahamas, B.W.I. / July 23,1951 / C. & P. Vaurie”; (b) TB# 13783.; (AMNH). PARATYPE (female) labeled (a) “South Bimini Isl. / Bahamas, B.W.I. / August 18,1951 / C. & P. Vaurie”; (b) TB # 14177.; (AMNH). PARATYPE (female) labeled (a) “South Bimini Isl. / Bahamas, B.W.I. / July 5,1951 / C. & P. Vaurie”; (b) TB # 14178”.; (AMNH). Two PARATYPES (female) (CMNC) labeled (a) “Guanahacabibes / Pen.,P.R.Cuba / July 3-4,1956 / C.&P.Vaurie”; (b) TB#’s 14181, 14182. PARATYPE (female) labeled (a) “BAHAMAS: ANDROS ID. / Fresh Creek, Androsia / Factory, 26.IV.1994-006 / R.S. Anderson beating / interior dry coppice”; (b) TB # 14684.; (CMNC). Two PARATYPE (female) (CMNC) labeled (a) “BAHAMAS: ANDROS ID. / London Ridge, 2.7 mi. N., / 0.8 mi. E., Forfar Field / Stn., 28.IV.1994-011, / R.S. Anderson, high / interior coppice beat”; (b) TB #’s 14682, 14683. Two PARATYPES (male) and one (female) (CMNC) labeled (a) “BAHAMAS: ANDROS ID. / London Ridge, 2.7 mi. N., / 0.8 mi. E., Forfar Field / Stn., 30.IV.1994-012, / R.S. Anderson, high / interior coppice”; (b) TB#’s 14685, 14686, 14687. Three PARATYPES (male) and one (female) (ZMHB) labelled (a) “Hist.-Coll (Coleoptera) / Nr. 46143 / Bolitophagus spec. / Cuba, Muller / Zool. mus. Berlin”; (b) TB #’s 14758, 14759, 14760, 14761. Seven PARATYPES (unknown sex) labeled (a) “Liho? Del Infierno? / Agosto 15/28”; (b) “Field Mus. Nat. His. / 1966 / A. Bierig Colln. / Acc. Z - 13812”; (c) TB# 14763, 14765, 14766.; (FMNH). Two PARATYPES (unknown sex) labeled (a) “Rm 14, Vinales / Agosto 14/28”; (b) “Field Mus. Nat. His. / 1966 / A. Bierig Colln. / Acc. Z - 13812”; (c) TB# 14764; (FMNH).

#### Diagnosis.

*Wattius viatorus* can be separated from the other West Indian members of the genus based on the following character combination: flight wings fully developed, meso- and metacoxae separated by more than mesocoxal width; pronotal horn strongly produced, apex expanded and bifurcated in males; femora lacking smooth rounded callosities; outer margins of tibia lacking distinct rows callosities, apical spine present on all tibia in males.

#### Description

**(Male).** Length 4.4–6.4 mm, width 1.8–2.6 mm (n = 44 specimens). Body, excepting antennae, eyes, underside of head, scutellum, tarsi, and coxae generally coated with thin shellac, often capturing debris on surface. Color ferruginous to black. **Head**: Frons and clypeus with dense deep foveae, somewhat shallower on clypeus, each fovea with one decumbent scale-like setae near center. Rounded setose tubercle lacking minute pit at apex present above eye, setae curved towards tubercle apex; tubercles absent between apex of eye and frontoclypeal margin. Frontoclypeal suture distinct, deeply impressed; clypeus with sharp lip along anterior margin, margin straight. Epistoma between eye and clypeus raised, rarely with one or two low tubercles weakly indicated. Deep impression present around eye from epistoma to apex. Eye reniform; emarginate at epistoma anteriorly, ventral lobe larger than dorsal, with micro-granulate and punctate triangular callus posterior to middle of eye. Labrum with transverse medial ridge, long golden setae present from ridge to anterior margin on dorsal surface, margin straight with setae on vertical surface. Mandible bifid at apex; maxillary palp four segmented, apical segment securiform; mentum trapezoidal, widest at anterior margin, medial longitudinal ridge present, strongest near anterior margin. Antenna with distinct three segmented club, club lighter than preceding segments and tomentose, antennomeres 10 and 11 fused, with sinus visible near lateral edges; antennomere 3 approximately 1.3× length of antennomere 4, antennomeres 4–8 subequal in length. **Prothorax**: Pronotal disc weakly convex, widest anterior to middle; densely, nearly confluently, deeply foveate, each fovea with one decumbent scale-like setae; densely tuberculate submedially, each tubercle bearing apical minute pit and covered in scale- like setae curved towards apex; anterior fourth of pronotum giving rise to raised medial horn, horn gradually sloping towards head, strongly expanded and either distinctly bifid or weakly medially emarginate in apical third of length; posterior fourth of pronotum with slight medial depression, lacking tubercles, near scutellum; lateral margin distinct and crenulate; anterior apices strongly produced and acute, posterior apices acute, not projecting. Hypomeron densely deeply foveate, each fovea with one decumbent scale-like setae. Prosternum anterior to coxa approximately as long as coxal cavity, medially nearly level with prosternal process; prosternal process raised between coxa, apex acute, projecting behind coxa. **Pterothorax**: Wings fully developed. Elytron parallel sided to posterior fourth, before sharply sloping and tapering caudad; stria weakly indicated by deep rounded punctures, interstria with somewhat regularly spaced tubercles and decumbent scale-like setae, tubercle structure as described for those on head and pronotum; 4th, 7th, and 10th interstria with tubercles forming weak costae, tubercles between 4th and 7th interstria and elytron suture occasionally forming irregular transverse costae. Scutellum glabrous and impunctate, ~1.4× wider than long, U- to approximately pentagonal in shape. Mesoventrite short, sparsely setose, distinctly emarginate behind prosternal process and forming submedial ridges anterior to mesocoxal cavities, mesocoxal cavities open. Metaventrite long, separating meso- and metacoxal cavities by more than mesocoxal cavity length, transversely rugose, sparsely setose with decumbent scale-like setae, moderately shallowly punctate around base of setae. All other ventrites on the pterothorax micro-granulate, often obscured by shellac, with decembent scale-like setae. **Legs**: Mesotrocantin exposed; femora lacking spines or other protrusions, sculpturing finely transversely rugose, lacking callosities, decumbent scale-like setae emerging from shallow folds throughout; tibia clothed in decumbent scale-like setae, outer margins lacking distinct rows of elongate smooth callosities, inner apical margin with socketed spurs vestigial to absent at base of small apical spine, patch of golden setae present on apical spines of all tibia; tarsal formula 5-5-4, venter of distal tarsomere on all legs with sparse golden setae, venter of all other tarsomeres clothed with dense long golden setae. **Abdomen**: Ventrites clothed in sparse decumbent scale-like setae, base of setae set in moderately sized punctures; abdominal intercoxal process wider than prosternal process, anterior margin straight to weakly rounded; intersegmental membranes concealed; ventrite 5 lacking submarginal groove; abdominal defensive reservoirs present; sternite viii weakly sclerotized and setose, deeply medially emarginate, emargination V-shaped; parameres fused, sharply acuminate to apex and weakly curved ventrad.

**Female.** Similar to male, but lacking apical tibial spine and horn not as strongly expanded and/or bifid at apex.

#### Distribution.

Cuba, Bahamas: South Bimini and North Andros Islands. Label data indicates that specimens have been collected between sea level and 20 meters in elevation.

#### Etymology.

The species epithet is a noun in apposition from the Latin *viator*, meaning traveler or tourist, due to the distribution of the species on multiple islands considered to be vacation destinations.

## Supplementary Material

XML Treatment for Wattius cucullatus

XML Treatment for Wattius asperulus

XML Treatment for Wattius andersoni

XML Treatment for Wattius emmabaconae

XML Treatment for Wattius viatorus
